# Iliac Telangiectatic Osteosarcoma - A Rare Presentation and Diagnostic Pitfall: A Case Report

**DOI:** 10.5704/MOJ.2011.034

**Published:** 2020-11

**Authors:** YC Khaw, JK Wong, Y Sahran, MZ Nor-Azman, WI Faisham

**Affiliations:** Department of Orthopaedics, Universiti Sains Malaysia, Kubang Kerian, Malaysia

**Keywords:** telangiectatic, osteosarcoma, iliac, biopsy, histopathology

## Abstract

Telangiectatic osteosarcoma is a rare variant of osteosarcoma and can be easily misdiagnosed as aneurysmal bone cyst. We report an atypical case of iliac telangiectatic osteosarcoma in a young healthy female, who presents with painful slow growing expansile lytic septate lesion in the left hemipelvis, which is initially treated as aneurysmal bone cyst. The diagnosis of aneurysmal bone cyst is made after histopathological examination of core needle biopsy. Her condition became unstable and massive bleeding is noted at the lesion site after sclerosant injection. She undergoes emergency hemipelvectomy and eventually the biopsy turns up to be telangiectatic osteosarcoma. Our case highlights that core needle biopsy is not useful in making diagnosis for iliac telangiectatic carcinoma. Hence, an open biopsy should be carried out in our case.

This case also emphasises on careful evaluation for malignancy which is mandatory because bleeding from pelvis after an unsuitable treatment can be grave, to the extent that major amputation hemipelvectomy is an option. Even though telangiectatic osteosarcoma has the same prognosis and treatment with conventional osteosarcoma, the outcome of delayed treatment for telangiectatic osteosarcoma is not good due to the dilemma in establishing an early correct diagnosis.

## Introduction

Telangiectatic osteosarcoma (TOS) of ilium is very rare. Pelvic TOS forms only 3.1% of all TOS1. The radiographic and histological features of Aneurysmal bone cyst (ABC) is often indistinguishable from TOS, which poses as a challenge to establish an early diagnosis^2^. Delay in the definite treatment for TOS carries a poor outcome. We reported an uncommon case of iliac TOS in an otherwise healthy 18-year-old female, which was initially treated as ABC. She had to undergo an urgent lifesaving hemipelvectomy after her general condition became unstable due to massive bleeding from the sclerosant injection site of left pelvis. The diagnosis of TOS was established through histopathologic examinations (HPE) following hemipelvectomy.

## Case Report

A healthy 18-year-old Malay female presented with slow growing locally painful swelling at left hip for three months. The pain was aggravated by movement, worse at night, and was relieved by taking painkiller. She walked with a limping gait. The swelling gradually enlarged for three months until she was completely bed ridden before being transferred to our hospital. Her appetite was reduced where she lost 5kg of weight within the three months. She denied any recent trauma, fever, family history of cancer and other joint pain or swelling.

Our examination revealed a diffuse swelling at the left iliac region, which was tender, warm, hard in consistency, not mobile and not fluctuant. Her left hip range of movement was limited due to pain. Neurovascular assessment of the left lower limb was normal. Patient was afebrile throughout her illness. All blood investigation was normal except an increase in the level of lactate dehydrogenase (733 U/L).

**Fig. 1:**
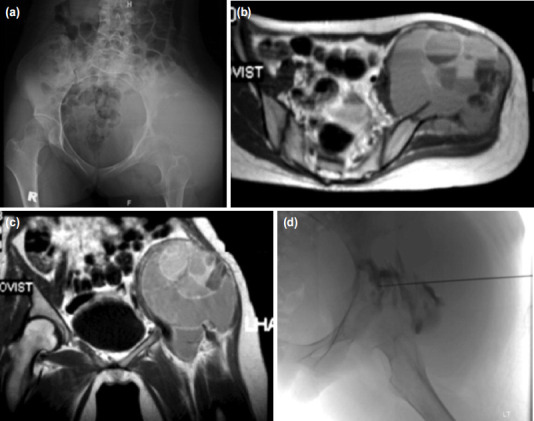
(a) Plain radiograph of pelvis showing a lytic lesion at left iliac region, without involvement of the left acetabulum and a narrow zone of transition with soft tissue shadow extension. (b) MRI pelvic axial view and (c) coronal view revelved a well demarcated lesion at left iliac with multiple fluid-fluid levels. Gluteal and iliopsoas muscle were pushed by expansile ilium and no evidence of infiltration. (d) Sclerosing injection to the pelvic.

**Fig. 2:**
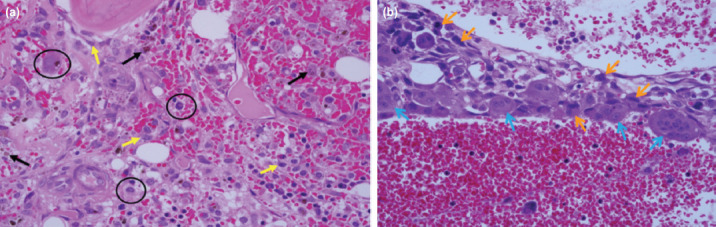
(a) Histopathological assessment of core needle biopsy revealed cystic spaces contain blood with the presence of macrophages (O), hemosiderin laden macrophages (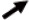) and reactive fibroblasts (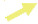), but no any atypical sarcomatous cell was seen. (Haematoxylin and eosin, x40). (b) Histopathological assessment from left hemipelvectomy amputated part showed septum which is lined by atypical sarcomatous cells (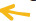) admixed with multinucleated giant cells (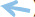) and cystic space containing blood (Haematoxylin and eosin, x 40).

Plain radiograph of pelvis (Fig. 1a) showed a lytic lesion at left iliac region with narrow zone of transition. There was soft tissue shadow extension and no involvement of the left acetabulum. Chest radiograph and CT thorax was normal. MRI (Fig. 1b and c) showed a well demarcated lesion with multiple fluid-fluid levels at left ilium which was suggestive of ABC. The gluteal and iliopsoas muscles were pushed by an expansile ilium with no evidence of infiltration. HPE result of left iliac core needle biopsy (Fig. 2a) showed cystic spaces contain blood with the presence of macrophages, hemosiderin laden macrophages and reactive fibroblasts, but no atypical sarcomatous cell was seen. The findings were consistent with ABC.

**Fig. 3:**
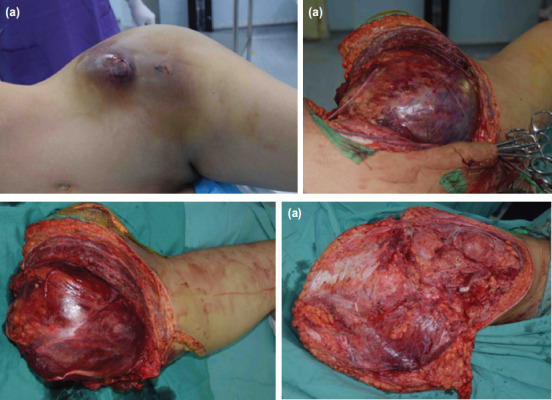
(a) Swelling at left iliac region post percutaneous sclerosing injection, (b,c) intra-operative photo showed emergency left hemipelvectomy amputated with clear margin. (d) Posterior flap hemipelvectomy was performed.

Percutaneous sclerosing injection of the left iliac lesion (Fig. 1d) was done as a treatment for ABC. Post procedure we noted blood oozing from the injection site. The site of injection was sutured, and bleeding stopped. Patient also developed popliteal vein thrombosis due to prolonged immobilisation, which was treated with subcutaneous enoxaparin sodium 40mg twice daily therapeutic dose. On day 10 post sclerosing injection, patient still had active bleeding from the left iliac injection site. She developed tachycardia with pulse rate of 110-120 and the blood pressure was 90/60mmHg. Haemoglobin level dropped to 6g/dL. Coagulation profile result was normal. Emergency hemipelvectomy with posterior flap was performed for the patient (Fig. 3). Intra-operative tissue was sent for HPE (Fig. 2b); results revealed lesion composed of multiple cystic spaces containing blood which were separated by irregular septa and lined by a mantle of atypical sarcomatous cells admixed with multinucleated giant cells. These atypical sarcomatous cells are pleomorphic, hyperchromatic and have an irregular round to oval nuclei. A diagnosis of left iliac TOS was made. Her hemipelvectomy operation scar healed after three weeks and she was on monthly regular follow-up. Five months after operation, she developed left psoas muscle and lung metastasis. Despite chemotherapy, she eventually succumbed to disseminated metastases, obstructive uropathy and bowel obstruction.

## Discussion

Even though osteosarcoma is the most common primary malignant bone tumour, TOS is a very rare variant, accounting for only 2 to 12% of all osteosarcoma^2^. TOS commonly occurs in metaphysis of long bones. It frequently involves femur, followed by tibia and humerus2. Iliac TOS is unique because of its rarity. The incidence of telangiectatic osteosarcoma in pelvis only 3.1%1.

Our patient with iliac TOS posed a challenge to us in making a diagnosis due to its uncommon location and its resemblance to ABC. TOS is often indistinguishable from ABC on radiograph^2^. Thus, the diagnosis should be established through careful histological examination1. However, HPE is a major dilemma because core needle biopsy in our case is not helpful for us in differentiating ABC with TOS. The accuracy of core needle biopsy for TOS is very low. Yuan *et al* reported core needle biopsies done by surgeons showing a lower success rate in TOS (55.6%), but a higher success rate (95.4%) in conventional osteosarcoma^3^. The accuracy of TOS is low because some parts of TOS are resembling ABC features in HPE. In addition, obtaining tissue through core needle biopsy to diagnose TOS in a large area of pelvic lesion is just like ‘finding a needle in a haystack’. Therefore, an open biopsy should be carried out in our case.

Massive bleeding from left pelvis after sclerosant therapy is a major dilemma to us in managing this patient. Limb salvage surgery was not suitable for our patient as her unstable condition does not permit for long operative time. Furthermore, deep vein thrombosis carries high risk of embolism. Thus, we opted for emergency hemipelvectomy as a life-saving procedure4, and we were able to secure the iliac vein to prevent pulmonary embolism. However, hemipelvectomy was a very tough decision as previously she was an active young lady.

The prognosis of TOS was initially thought to be poor, but it is no longer true as current literature has shown that TOS and conventional osteosarcoma share the same treatment and prognosis with the survival rate of 60% at 10 years^5^. However, the prognosis in our patient with iliac TOS was not favourable as we encountered difficulties in establishing an early correct diagnosis. Early diagnosis in TOS with prompt treatment was imperative for disease-free survival^2^.

In conclusion, iliac TOS is often misdiagnosed as ABC. Therefore, awareness of this uncommon condition and high index of suspicion is important to establish early correct diagnosis. Open biopsy should be performed for iliac telangiectatic osteosarcoma if core biopsy result is doubtful.
